# Localized cardiac metabolic trajectories and post-infectious metabolic sequelae in experimental Chagas disease

**DOI:** 10.21203/rs.3.rs-2497474/v1

**Published:** 2023-01-20

**Authors:** Zongyuan Liu, Rebecca Ulrich, April L. Kendricks, Kate Wheeler, Ana Carolina Leão, Jeroen Pollet, Maria Elena Bottazzi, Peter Hotez, Fabian Gusovsky, Kathryn M. Jones, Laura-Isobel McCall

**Affiliations:** 1 Department of Chemistry and Biochemistry, University of Oklahoma, Norman, Oklahoma, United States of America; 2 Laboratories of Molecular Anthropology and Microbiome Research, University of Oklahoma, Norman, Oklahoma, United States of America; 3 Department of Biomedical Engineering, University of Oklahoma, Norman, Oklahoma, United States of America; 4 Southern Star Medical Research Institute, Houston, TX, United States of America; 5 Department of Biology, University of Oklahoma, Norman, Oklahoma, United States of America; 6 Department of Pediatrics, Baylor College of Medicine, Houston, TX, United States of America; 7 Department of Molecular Virology and Microbiology, Baylor College of Medicine, Houston, TX, United States of America; 8 Global Health Research, Eisai, Inc., Cambridge, MA, USA; 9 Department of Microbiology and Plant Biology, University of Oklahoma, Norman, Oklahoma, United States of America

## Abstract

Post-infectious conditions, where clinical symptoms fail to resolve even after pathogen clearance, present major health burdens. However, the mechanisms involved remain poorly understood. In Chagas disease (CD), caused by the parasite *Trypanosoma cruzi*, antiparasitic agents can clear *T. cruzi* but late-stage treatment does not improve clinical cardiac outcomes. In this study, we revealed differential metabolic trajectories of cardiac regions during *T. cruzi* infection, matching sites of clinical symptoms. Incomplete, region-specific, cardiac metabolic restoration was observed in animals treated with the antiparasitic benznidazole, even though parasites were successfully cleared. In contrast, superior metabolic restoration was observed for a combination treatment of reduced-dose benznidazole plus an immunotherapy (Tc24-C4 *T. cruzi* flagellar protein and TLR4 agonist adjuvant), even though parasite burden reduction was lower. Overall, these results provide a mechanism to explain prior clinical treatment failures in CD and to test novel candidate treatment regimens. More broadly, our results demonstrate a link between persistent metabolic perturbation and post-infectious conditions, with broad implications for our understanding of post-infectious disease sequelae.

## Introduction

Antimicrobial treatment, including antibacterials, antivirals, antifungals and antiparasitics, is a mainstay of therapeutic strategies against infectious agents, with treatment success often defined by metrics associated with pathogen clearance. However, as post-infectious irritable bowel syndrome, post-infectious Lyme arthritis, post-acute sequelae of non-persistent viruses, and many other conditions demonstrate, pathogen clearance does not always lead to symptomatic cure ^1,2,3^. While the pathogenesis of long COVID is still under study, some postulated mechanisms include symptom persistence and/or emergence even after viral clearance ^4^. The study of post-infectious sequelae, their mechanisms, and their treatment, has unfortunately lagged compared to the study of acute infection pathogenesis and treatment. Such a need is particularly clear in Chagas disease (CD): clinical trial data has demonstrated that antiparasitic treatment with the drug benznidazole (BNZ) is insufficient to prevent disease progression or mortality if administered late in the disease, even in patients showing undetectable parasite burden ^5^. This mechanism is distinct from parasitological treatment failure, with parasite persistence, which may result from a combination of the local nutritional environment, reduced prodrug activation via drug resistance mechanisms, and parasite dormancy ^6,7,8,9^. Thus, a better understanding of the mechanisms of BNZ treatment failure may inform CD drug development, but also help guide the development of new tools and new interventions for other chronic and post-infectious illnesses.

CD is caused by the intracellular protozoan parasite *Trypanosoma cruzi* ^10–12^. Approximately 6-8 million people are infected with *T. cruzi*, which causes more than 12,000 deaths per year ^13^. CD is endemic in Latin America but has become a global health issue due to immigration. Close to 70 million people worldwide are at risk of infection. After acute infection, patients without treatment usually progress to the chronic phase of disease. The chronic phase has four forms: the indeterminate form with no apparent clinical symptoms, the cardiac form, the digestive form, and the cardiodigestive form. The cardiac form is a major cause of morbidity and mortality, with symptoms of dilated cardiomyopathy, congestive heart failure, arrhythmias, cardioembolism, and stroke ^14^.

Immunoregulatory mechanisms are important processes for the control of the immune-mediated damage observed in chronic CD^15–17^. Studies comparing *T. cruzi*-specific immune responses in indeterminate patients to patients with cardiac disease have identified key aspects of the host immune response that correlate with disease severity. Indeterminate patients without overt clinical disease exhibit a mixed T_H_1/T_H_2/T_H_17 immune profile, with increased levels of the key cytokines IFNγ, IL-10, and IL-17 ^18–22^. In contrast, patients with clinical signs of cardiomyopathy have a predominantly T_H_1 immune profile, with increased levels of the pro-inflammatory cytokines IFNγ, IL-6, TNFα and IL-1β and very little IL-10 and IL-17 ^18–22^.

Building on the knowledge that a balanced immune response correlates with reduced disease symptoms, we developed a vaccine containing recombinant Tc24-C4 protein combined with a TLR4 agonist adjuvant ^23,24^. The Tc24 antigen is a *T. cruzi* flagellar calcium-binding antigen that is conserved across multiple discrete typing units of *T. cruzi*, and is expressed in extracellular trypomastigote and early intracellular amastigote stages ^25,26^. When used as an immunotherapy in experimentally infected mice, the vaccine increased levels of antigen-specific CD8^+^ cells as well as the key cytokines IFNγ, IL-10, and IL-17A ^24,27,28^. Importantly, combining this vaccine with BNZ treatment in a vaccine-linked chemotherapy strategy led to significantly reduced cardiac pathology while reducing the amount of BNZ necessary for efficacy ^27–29^. Together, these data suggest that through induction of a balanced T_H_1/T_H_2/T_H_17 immune response, the Tc24-C4 immunotherapy can overcome some limitations of BNZ treatment alone in Chagasic patients.

Immune responses and metabolism are tightly linked. In CD, gradients of parasite burden, metabolic alterations and immune responses were observed in the heart ^30,31^. Cardiac metabolic alterations were correlated to the degree of cardiac inflammation, and to serum profibrotic cytokine levels ^32^. These metabolic alterations were causally linked to *T. cruzi* infection outcomes, and metabolism-targeting strategies prevented acute CD mortality in mouse models ^33^. However, the impact of antiparasitic treatment and immunomodulatory strategies on local metabolic responses in chronic CD and in post-treatment recovery is currently unknown.

To address this gap, we combined in this study mass spectrometry-based metabolomics with three-dimensional modeling (“chemical cartography” ^30^) to investigate the spatial effects of infection, Tc24-C4 immunotherapy and BNZ on the cardiac metabolome and metabolic restoration post-treatment. Our results provide insights into mechanisms of treatment success and demonstrate the key role of cardiac metabolism in CD treatment success. Furthermore, our results represent a new paradigm into metabolic determinants of post-infectious sequelae.

## Methods

### Ethical approval

All animal studies were conducted in strict compliance with the 8^th^ Edition of The Guide for Care and Use of Laboratory Animals ^34^ and were approved by the Baylor College of Medicine Institutional Animal Care and Use Committee under assurance number D16-00475.

### *In vivo* experimentation - infection, treatments and endpoints

The *T. cruzi* H1 strain, transfected with the pTRIX2-RE9h plasmid containing the thermostable red-shifted firefly luciferase gene *PpyRE9h* ^35–37^, was grown on monolayers of the C2C12 mouse myocyte cell line (ATCC CRL-1772) in RPMI media supplemented with 5% fetal bovine serum and 1X Penicillin/Streptomycin (cRPMI) to propagate tissue culture trypomastigotes (TCT). Culture media containing TCT was collected, parasites were pelleted by centrifugation, washed once with sterile medical grade saline, then resuspended in sterile medical grade saline to 5 x10^4^ trypomastigotes per milliliter for infection. Female BALB/c mice (Taconic Biosciences, Inc) aged 6-7 weeks old were infected by intraperitoneal injection with 5,000 TCT in a final volume of 0.1 mL. Mice were monitored daily for morbidity and any mice that reached humane endpoints were euthanized. At 69 days post infection, mice were randomly divided into groups of 15 mice each and treated with 100 mg/kg BNZ (BNZ-only treatment mice, MedChem express) or 25 mg/kg BNZ (combination treatment mice) suspended in 5% dimethylsufoxide/95% HPMC solution (0.5% hydroxypropylmethylcellulose, 0.4% Tween 80, 0.5% benzyl alcohol) by oral gavage once-daily for 18 days. Mice in the combination treatment group then received 25 μg Tc24-C4 protein + 5 μg E6020 adjuvant in a stable squalene emulsion (Tc24-C4/E6020-SE) by subcutaneous injection twice, two weeks apart, at days 92 and 106 post-infection. Recombinant Tc24-C4 protein was expressed and purified according to previously published protocols. The TLR4 agonist adjuvant E6020 (Eisai, Inc) was formulated in a stable squalene emulsion (SE). Vaccine formulations comprised of the recombinant Tc24-C4 protein and E6020 in 100 μL of a 2% squalene emulsion in PBS 1x pH 7.4 were freshly prepared and mixed just before administration ^24^. Naive age matched mice and infected mice that were left untreated were included as controls. Blinding was not possible, given the need to ensure that the right mice received the right treatment. However, treatment and metabolomics analysis (see below) were performed by different investigators. Mice were humanely euthanized at 50, 75 (no-treatment timepoints), or 142 days post infection (DPI), and hearts were excised. The atria were removed in their entirety, then left and right ventricles were separated and divided into top and bottom sections. All samples were snap frozen on dry ice, then stored at −80°C until further analysis. Mouse parameters (weight, tissue weight, echocardiography, electrocardiography and immunological parameters) were analyzed using two-sided T-tests, assuming data normality, with FDR correction for multiple comparisons between treatment groups.

### *In vivo* experimentation - echocardiography and electrocardiograms

Data was acquired once per animal post-treatment. Mice were anesthetized by inhalation of 2-3% isoflurane delivered by precision vaporizer. Fur from the ventral thorax of anesthetized mice was removed with depilatory cream, then mice were positioned in dorsal recumbency on a temperature-regulated stage set at 37°C. Core body temperature and heart rate were monitored by rectal thermometer and Doppler electrocardiogram. Prewarmed ultrasound gel was applied to the thorax, and short axis images of the left ventricle were obtained from the left parasternal window with a Vevo 2100 imaging system (FujiFilms Visualsonics, Inc.). M-mode images were obtained at the papillary level to determine left ventricular chamber dimensions and wall thickness. Immediately after echocardiographic evaluation, mice were transferred to a Rodent Surgical Monitor (Mouse Monitor, Indus Instruments) to obtain Lead II electrocardiograms. M-mode images were analyzed using VevoLab software (Fujifilm Visualsonics) to measure left ventricular wall thickness and left ventricle chamber dimensions. Electrocardiogram tracings were analyzed using LabChart Pro software to measure conduction intervals and wave amplitudes.

### Splenocyte restimulation for immune evaluation

To prepare single cell splenocyte suspensions, spleens were mechanically dissociated through 70 μm cell strainers, red blood cells were lysed with ACK lysis solution (Gibco), then washed with RPMI supplemented with 10% fetal bovine serum (FBS), 1X Pen/Strep and L-Glutamine (cRPMI). Live cells were quantified using a Cellometer Auto 2000 and AOPI live/dead dye (Nexcelom), then adjusted to a final concentration of 1x10^7^ cells/mL in cRPMI. For each sample, 1x10^6^ live splenocytes were restimulated for 96 hours with 100 μg/mL recombinant Tc24-C4 protein or cRPMI (unstimulated) at 37°C, 5% CO_2_. As a positive control, splenocytes incubated for 6 hours with 20 ng/mL phorbol myristate acetate (PMA) (Sigma-Aldrich) and 1 mg/mL Ionomycin (Sigma-Aldrich) were included.

### Evaluation of cytokine-producing splenocytes

To quantify antigen-specific cytokine-producing CD4^+^ and CD8^+^ cells, cells were restimulated as described, with the addition of 4.1 μg/mL Brefeldin A (BD Biosciences) for the last 6 hours of incubation. Restimulated splenocytes were collected, washed with 1 X PBS, and stained with Live/Dead fixable blue viability dye, anti-CD3e Pacific Blue clone 145-2C11 (Biolegend), anti-CD4 Alexa Fluor^®^ 700 clone RM4-5 (eBioscience) and anti CD8a PerCP-Cy5.5 clone 53-6.7 (BD Bioscience). Splenocytes were then fixed with Cytofix/Cytoperm (BD Biosciences) and permeabilized following manufacturer instructions. Permeabilized splenocytes were stained with anti-IFNγ APC clone XMG1.2 (eBioscience), anti-IL-17A clone TC11-18H10.1 (Biolegend), anti-IL-2 Brilliant Violet 510 clone JES6-5H4 (Biolegend), anti-IL-4 PE-Cyanine7 clone BVD6-24G2 (eBioscience), and anti-TNFα PE clone MP6-XT22 (eBioscience). Samples were acquired on a LSR Fortessa Cell Analyzer (BD Biosciences) and 25,000 total events in a live gate were analyzed using FlowJo 10.8.1 software. To evaluate antigen-specific responses the percent of unstimulated cells was subtracted from cells stimulated with Tc24-C4 protein for each sample.

### Metabolite extraction

Extraction batches included equal representation of samples across groups, with one extract per tissue segment per mouse. Tissue was extracted, as in our prior work ^30^, using LC-MS-grade water (Fisher Optima) at a constant ratio of 50 mg of tissue in 8,000 μL of water, followed by 3 min homogenization at 25 Hz in a Qiagen TissueLyser with a 5-mm steel ball. A portion of the homogenate was stored at −80°C for subsequent DNA extraction. LC-MS-grade methanol (Fisher Optima) spiked with 4 μM sulfachloropyridazine was added to the rest of the homogenate to achieve a final 50% methanol concentration, then homogenized again at 25 Hz for 3 min. After the homogenate was centrifuged for 10 min at 14,980 x g at 4°C, the supernatant was dried in a Savant SPD111V (ThermoFisher Scientific) SpeedVac concentrator overnight. For organic extraction, 3:1 (v:v) dichloromethane (Fisher Optima)–to–methanol solvent mixture spiked with 4 μM sulfachloropyridazine was added to the insoluble fraction from the previous step and re-homogenized at 25 Hz for 5 min. Extracts were centrifuged for 10 min at 14,980 x g at 4°C, and air-dried overnight. Both extracts were stored at −80°C until LC-MS analysis.

### DNA extraction

DNA extraction was performed on the water homogenate from the ventricles using the Quick-DNA Miniprep Plus Kit for solid tissues (Zymo Research), with some deviations as follows: 95 μL of the water homogenate from the metabolite extraction was used during the initial protocol step; proteinase K digestion was 1 h in duration. Insufficient material remained from the metabolite extraction to enable DNA extraction for the atria.

### qPCR

qPCR was performed as previously described ^30^ using primers ASTCGGCTGATCGTTTTCGA and AATTCCTCCAAGCAGCGGATA to amplify *T. cruzi* ^38^ and TCCCTCTCATCAGTTCTATGGCCCA and CAGCAAGCATCTATGCACTTAGACCCC to normalize to mouse TNFα reference gene ^39^ . Each DNA sample was diluted to 180 ng/μL for analysis, and analyzed in duplicate with each primer, using PowerUp SYBR Green Master Mix (Thermo Fisher). A serial dilution was performed from DNA extracted from tissues spiked with known parasite numbers, diluted with DNA from uninfected mice, to generate a standard curve. Values below the limit of detection were marked as 0 parasite burden in the analysis. Non-parametric two-sided tests were used for analysis, with no assumption of normality.

### LC-MS/MS data acquisition

Prior to LC-MS/MS data acquisition, dried aqueous and organic extracts were resuspended with 50% methanol (Fisher Optima; LC-MS grade) spiked with 2 μM sulfadimethoxine (Sigma-Aldrich) as internal standard, then both extracts were combined. Data was acquired in random order for each sampling site, with blanks and pooled quality controls every 12 injections. Injection volume for each sample was 30 μL. Data acquisition was performed a single time per sample. Instrument was calibrated using Calmix calibrant (Thermo Scientific). LC separation was performed on a Thermo Scientific Vanquish UHPLC system with 1.7 um 100 Å Kinetex C8 column under 40°C. Mobile phase A was water with 0.1% (v:v) formic acid and mobile phase B was acetonitrile with 0.1% (v:v) formic acid. The LC gradient was: 0-1 min, 2% B; 1-2.5 min, ramp up linearly to 98% B; 2.5-4.5 min, hold at 98% B; 4.5-5.5 min, ramp down to 2% B; 5.5-7.5 min hold at 2% B. MS/MS analysis was performed on a Q Exactive Plus (ThermoScientific) mass spectrometer. Ions were generated by electrospray ionization and MS spectra acquired in positive ion mode (**Supplementary Table 1**). Instrumental performance was assessed throughout data acquisition using a standard mix of 6 known compounds at run start, run end, and every 100 samples.

### LC-MS/MS data processing

3D heart model was purchased from 3DCADBrowser.com (http://www.3dcadbrowser.com/). MS Raw data were converted to mzXML format by MSConvert software ^40^. Converted mzXML files were processed in MZmine version 2.5.3 (see **Supplementary Table 1** for parameters). Data were filtered to obtain MS1 scans that were present in at least three samples and were associated with MS2 spectra for annotation, with good extracted ion chromatogram peak shape. Blank removal was performed, with a minimum threefold difference between blank and samples required for a feature to be retained. No other exclusions were performed. No samples were excluded. Total ion current (TIC) normalization was performed in Jupyter Notebook using R version 3.6.1 (http://jupyter.org). Principal coordinates analysis (PCoA) and PERMANOVA analyses were performed in QIIME2 and visualized in EMPeror ^41,42^.

Non-parametric two-sided tests were used for analysis, with no assumption of normality. Distances were compared using non-parametric Kruskal-Wallis tests with post-hoc Dunn’s test, FDR-corrected. For time series data, Mann-Whitney two-sided U test with FDR correction was performed to identify features with FDR-corrected p<0.05 for naïve VS infected without treatment at at least one of the 50, 75 or 142 days post infection timepoints, followed by random forest analysis to rank their importance in differentiating between groups, using 1000 trees ^43^. Features with Variable Importance>2.1 were retained. The impact of treatment was likewise assessed using random forest analysis. First, a random forest classifier comparing uninfected and infected untreated groups at 142 days post-infection was built using 1000 trees. Features with Variable Importance>2.1 were retained. To identify metabolites restored by the different treatment regimens, the original metabolite feature table was filtered to this subset and Mann-Whitney two-sided U test with FDR correction performed between each treatment group to the naive group. Features with FDR-corrected p>0.05 represent features restored by these treatments.

The overlap between features perturbed at the different timepoints or restored by the different treatments was visualized using UpSet plots ^44^ version 0.6 in python 3.8. The total size of each set is represented on the left barplot. Intersections are represented by the bottom plot, and their occurrence is shown on the top barplot. Barplot colors indicate metabolite superclass as determined by Classyfire^45^, implemented in MolNetEnhancer^46^. Boxplots represent median, upper and lower quartiles, with whiskers extending to show the rest of the distribution, except for points that are determined to be “outliers” by being beyond the interquartile range +/− 1.5 times the interquartile range.

Feature-based molecular networks were created using the Global Natural Products Social Molecular Networking platform (GNPS) ^47,48^. The parameters for the spectra and library searches were: 0.02 Da for both precursor ion mass tolerance and MS/MS fragment ion mass tolerance, ≥0.7 cosine score, ≥4 matched peaks, and 100 Da maximum analog search mass difference. All reported annotations, which were collected by an automated script from the GNPS output (https://github.com/camilgosmanov/GNPS) ^49^, are within 10 ppm, at Metabolomics Standards Initiative confidence level 2 ^50^.

Figure 3A was created with BioRender.com.

Pairwise correlation between disease parameters and restored or not-restored metabolites was calculated using pandas.DataFrame.corr in Pandas python package, with Spearman method. FDR-corrected p values were obtained using statsmodels.stats.multitest.fdrcorrection from the statsmodels 0.14.0 python package. Correlation data was visualized using Cytoscape version 3.9.1 ^51^.

Fisher’s exact test was calculated using https://www.socscistatistics.com/tests/fisher/default2.aspx.

### Data availability

Data has been deposited in MassIVE, accession number MSV000087427. Molecular networks can be accessed at https://gnps.ucsd.edu/ProteoSAFe/status.jsp?task=dd1ef14c8a964bfd8843da96aa957d89 (feature-based molecular network) and https://gnps.ucsd.edu/ProteoSAFe/status.jsp?task=3fefbc8549604d34954aba2a95ec79df (MolNetEnhancer ^46^).

### Code availability

Representative code has been deposited in GitHub: https://github.com/zyliu-OU/McCall-Lab/tree/main/03172021.

## Results

### Infection-induced metabolic perturbations are highly localized and established early post-infection

**Figure 1.**
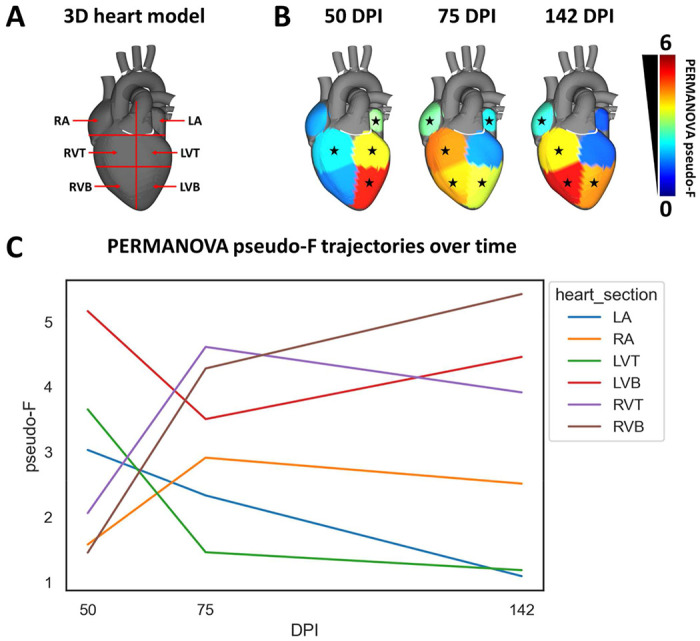
Infection-induced metabolic perturbations are highly localized spatially and temporally. (A) Heart sections analyzed. RA, right atrium. LA, left atrium. RVT, right ventricle top. RVB, right ventricle bottom. LVT, left ventricle top. LVB, left ventricle bottom. (B) PERMANOVA pseudo-F for PCoA distances between naïve and infected at 50, 75, and 142 days post infection (DPI). *, p-value <0.05 by PERMANOVA. (C) Site-specific PERMANOVA pseudo-F trajectories over time. N=15 mice per group and per position.

Given the specific localization of CD lesions ^52,53^, we assessed the impact of infection on metabolism and the trajectories of infection-induced metabolic perturbations between six heart segments: left atrium, right atrium, top half of the left ventricle free wall, bottom half of the left ventricle free wall, top half of the right ventricle free wall, and bottom half of the right ventricle free wall (Fig. 1A, N=15 mice per group and per position). Strikingly, different cardiac regions demonstrated different metabolic trajectories, which correlated with sites of clinical CD pathology (Fig. 1B, Fig. 1C, **Supplementary data 1**). The impact of infection on overall metabolism in the atria was minor at early timepoints post-infection (left atrium, PERMANOVA p=0.014, pseudo-F statistic=3.03; right atrium, PERMANOVA p=0.12, pseudo-F statistic = 1.58), and either decreased over time (left atrium, Kruskal-Wallis (KW) with post-hoc Dunn’s test, FDR-corrected, p= 2.68e-18 for distances between infected and uninfected samples at 50 days vs 142 days) or remained overall unchanged (right atrium, KW with post-hoc Dunn’s test, FDR-corrected, p=0.77 for distances between infected and uninfected samples at 50 days vs 142 days). Similar to the left atria, the overall metabolome at the top of the left ventricle was only significantly affected by infection at early timepoints (PERMANOVA p=0.005 pseudo-F statistic=3.65 at 50 days post-infection; p>0.05 at 75 and 142 days post-infection). In contrast, the overall metabolome at the bottom of the left ventricle was significantly affected at all timepoints (PERMANOVA p=0.003 pseudo-F statistic= 5.16 at 50 days post-infection; p=0.002 pseudo-F statistic=3.50 at 75 days post-infection; p=0.001 pseudo-F statistic=4.46 at 142 days post-infection), though metabolism partially renormalized over time (KW with FDR-corrected post-hoc Dunn’s test p=9.92e-17 for distances between infected and uninfected samples at 50 days vs 142 days). With regards to the right ventricle, the overall metabolome was significantly and persistently affected at all timepoints at the top of the right ventricle (PERMANOVA p<0.05, pseudo-F statistic=2.061 at 50 days post-infection, pseudo-F statistic=4.61 at 75 days post-infection and pseudo-F statistic=3.91 at 142 days post-infection), and from 75 days onwards at the bottom of the right ventricle (PERMANOVA p<0.05 pseudo-F statistic=4.28 at 75 days post-infection and pseudo-F statistic=5.43 at 142 days post-infection). Strikingly, the magnitude of metabolic perturbation was highest in the bottom segments of both the left and right ventricles at our chronic 142 days post-infection timepoint, matching with the sites of CD damage in patients ^53^. This localized metabolic perturbation to the heart apex contrasts with our observation of comparable parasite load between ventricle sites at 142 dpi (KW p>0.05, FDR-corrected, Fig. 3BF), but concur with our prior observations of disconnect between parasite tropism and location of metabolic perturbation ^31,33^.

**Figure 2.**
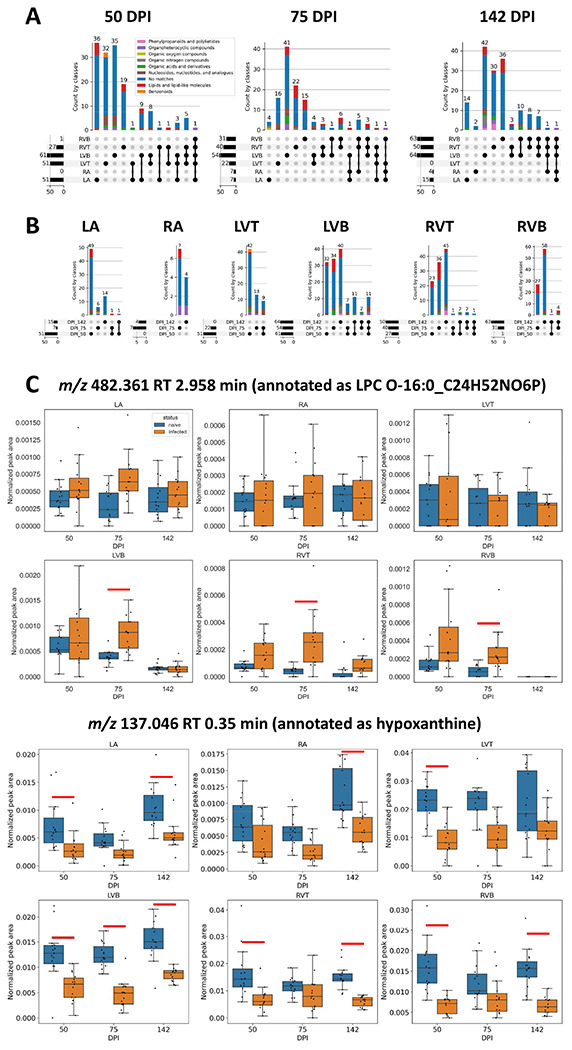
Impact of infection progress on individual metabolites. (A) UpSet plot demonstrating limited overlap between infection-impacted metabolites at each heart section, for each timepoint post-infection. Bars are colored by superclass from ClassyFire annotation, as implemented in MolNetEnhancer ^46,45^. Dark circles represent intersections between groups, with the size of that intersection on top of the colored bar graph. Total number of features impacted by infection at each sampling site is represented on the left barplot. (B) UpSet plot demonstrating limited overlap between infection-impacted metabolites at each timepoint, for each of the heart sections. (C) Representative metabolites locally impacted by infection over time. Red line, p-value <0.05 by Mann-Whitney U Test, FDR-corrected. N=15 mice per group and per position.

The specific metabolites perturbed by infection were mainly site-specific (Fig. 2A) and timepoint-specific (Fig. 2B). This indicates localized, site-specific responses to *T. cruzi*, rather than baseline differences in metabolism, since there was considerable overlap across sites and timepoints in terms of metabolite absence/presence (**Supplementary figure 2**). Strikingly, the greatest overlap between infection-perturbed metabolites across timepoints was observed in the left ventricle bottom, which may indicate a stronger role between pathogenesis and metabolism at this site. Overall infection-perturbed metabolite features include purines, amino acids, and multiple lipids (Fig. 2C, **Supplementary Table 2**). Purines were consistently depleted across sections and at all timepoints. In contrast, *m/z* 307.084 RT 0.362 min (annotated as oxidized glutathione) was only significantly elevated by infection in the left ventricle bottom, whereas it was detected but not significantly elevated in the right ventricle. Glycerophosphocholines such as *m/z* 482.361 RT 2.958 min were elevated by infection most strongly at 75 days post-infection, in the left and right ventricle bottoms and in the right ventricle top.

### Standard-of-care BNZ does not fully restore metabolic alterations

**Figure 3.**
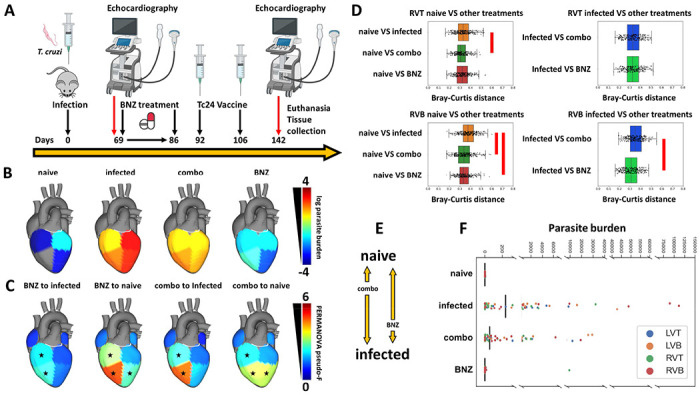
Standard-of-care BNZ does not fully restore metabolic alterations while vaccine-linked chemotherapy showed greater improvement. (A) Treatment timeline. (B) Median parasite burden in each group (log scale). Right ventricle bottom values in naive mice not displayed due to log scale (median of zero). (C) PERMANOVA pseudo-F at 142 days post infection for PCoA distances between naïve mice and the different treatment groups or between infected untreated mice and the different treatment groups. (D) PCoA distances between experimental groups at 142 days post-infection. In particular, BNZ-treated samples were not as distant from infected samples as combo-treated samples at RVB. Red bars, KW with post-hoc Dunn’s test, FDR-corrected p<0.05. RVT, right ventricle top; RVB, right ventricle bottom. N=15 mice per group and per position. (E) Diagram of restoration of metabolic status based on distance analysis from panel D. (F) Parasite burden in individual samples.

As the BENEFIT clinical trial demonstrated ^5^, BNZ treatment is insufficient to prevent adverse clinical outcomes in late-stage CD patients, even with successful parasite clearance. The mechanisms responsible for this lack of efficacy are however insufficiently characterized. Based on our findings of tissue location-specific adverse metabolic trajectories during infection (Fig. 1C) and our prior observations of relation between experimental CD severity and magnitude of metabolic perturbation ^31–33^, we hypothesized that the lack of clinical efficacy of BNZ in late-stage CD may be due to an inability to restore infection-induced metabolic alterations at specific cardiac locations. To test this hypothesis, mice were treated with BNZ standard-of-care (100 mg/kg BNZ for 18 days, beginning 69 days post-infection) (Fig. 3A, N = 15 mice per position and per group). Electrocardiographic data and echocardiographic data were acquired post-treatment, and animals were euthanized at 142 days post-infection. We assessed 50 phenotypic indicators of disease severity, including heart weight, liver weight, body weight, echocardiographic parameters, electrocardiographic parameters and immunological parameters. Thirteen of these parameters showed significant differences between infected and uninfected animals (T test, uncorrected p<0.05). Only half of these parameters were restored by BNZ treatment (Table 1), even though parasite burden in BNZ-treated animals was no longer significantly different from uninfected animals at all ventricle sites (p>0.05, KW with post-hoc Dunn’s test, FDR-corrected), and differed significantly from untreated animals (p<0.05, KW with post-hoc Dunn’s test, FDR-corrected) (Fig. 3BF). Thus, our biological model indicates only partial phenotypic efficacy of standard BNZ-treatment, modeling the clinical situation ^5^.

Strikingly, of the two sites most significantly impacted by infection at 142 days post-infection, the bottom of the left and right ventricles (Fig. 1B), BNZ only re-normalized metabolism at the bottom of the right ventricle (KW with FDR-corrected post-hoc Dunn’s test p = 1.44e-07) (Fig. 3CD). Nevertheless, metabolism remained significantly different from uninfected animals at this site (Fig. 3CD, PERMANOVA p< 0.05), indicating that BNZ alone, followed by 56 days of recovery, is unable to fully lead to metabolic restoration. We had one outlier in the BNZ treatment group with high parasite burden in one sampling site only. However, conclusions were not affected by this outlier mouse ( **Supplementary data 1**).

### Improved metabolic restoration with combination BNZ and immunotherapy treatment compared to BNZ-alone treatment

Given the association between CD and immune responses ^18^ and the poor tolerability of standard BNZ treatment regimens in humans ^54,55^, we then assessed whether an experimental regimen consisting of a combination of reduced-dose BNZ (25 mg/kg for 18 days) followed by two doses of a therapeutic vaccine, Tc24 C4 (25 μg at days 92 and 106 post-infection), could provide a phenotypic and metabolic benefit. This combination treatment did not significantly reduce parasite burden (p>0.05, KW with post-hoc Dunn’s test, FDR-corrected, Fig. 3BF). However, it successfully restored 3 of our phenotypic indicators of disease, all immunological, that BNZ-alone treatment had failed to improve. Five disease indicators were not-restored by the combination treatment, two of which also failed to be restored by BNZ-alone treatment (liver weight to body weight ratio and P wave amplitude, Table 1). The combination treatment led to improved cardiac metabolic restoration compared to BNZ alone in the right ventricle bottom (increased distance to infected samples in combo-treated animals, KW with FDR-corrected post-hoc Dunn’s test p =7.43e-08 ) (Fig. 3CD, **Supplementary data 1**), and reduced the distance to uninfected samples in the right ventricle top and bottom (KW with FDR-corrected post-hoc Dunn’s test p = 8.018e-03 for RVT and p = 3.58e-07 for RVB), though it was unable to fully restore metabolism (Fig. 3DE). These findings indicate that parasite clearance alone is insufficient to improve metabolism, and that the inability of BNZ to fully restore metabolism is not due to lingering parasite fragments. These results also provide a mechanism to explain the failure of BNZ in late-stage CD treatment and indicate that alternative treatment regimens, even those that do not fully clear parasites, may instead be desirable and can show improved metabolic restoration.

**Figure 4.**
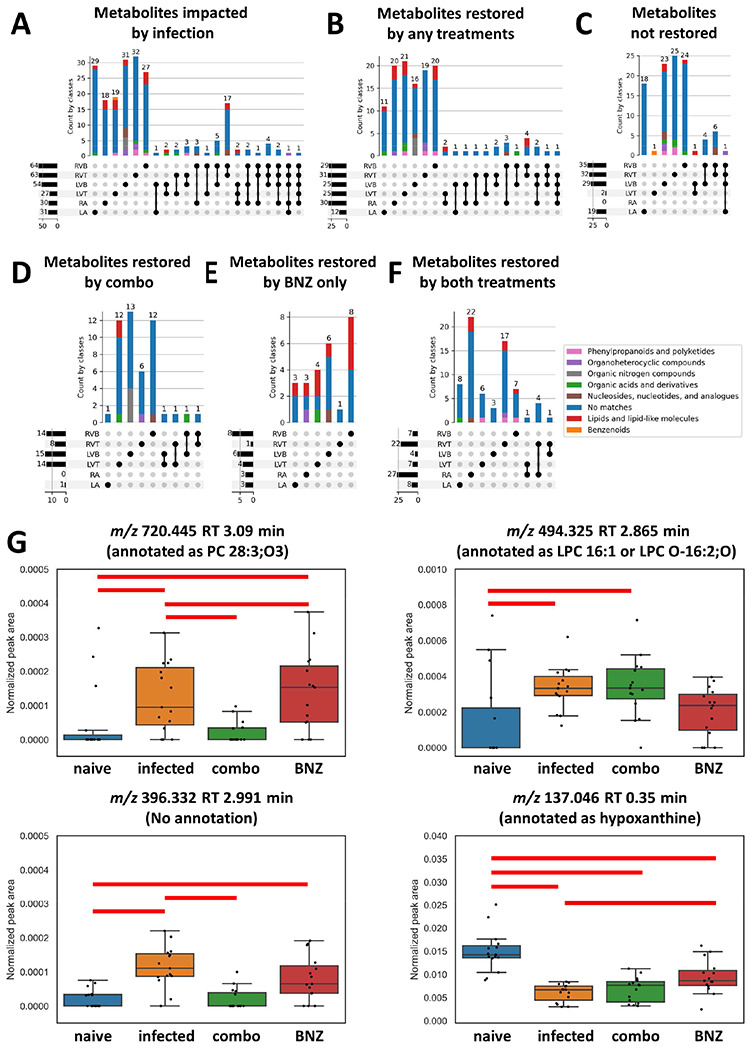
UpSet plot analysis demonstrating that BNZ + Tc24 vaccine combination treatment restored more infection-perturbed metabolites than BNZ-only treatment. All metabolites in panel A came from random forest analysis between naïve and infected mice at 142 DPI. Bars are colored by superclass annotation generated in Classyfire, as implemented in MolNetEnhancer. (A) Metabolites impacted by infection. (B) Metabolites restored by any treatment (BNZ-only or combo treatment (BNZ+Tc24-C4/E6020-SE)). (C) Metabolites not restored by any treatment. (D) Metabolites only restored by BNZ + Tc24 vaccine treatment. (E) Metabolites only recovered by BNZ treatment. (F) Metabolites commonly restored by both treatments. RA, right atrium; LA, left atrium; LVT, left ventricle top; LVB, left ventricle bottom; RVT, right ventricle top; RVB, right ventricle bottom. N=15 mice per group and per position. (G) Representative metabolites restored by different treatments. red line, p-value <0.05 by Mann-Whitney U Test between two groups, FDR corrected.

To determine the specific metabolic pathways that fail to be restored by the different treatments or can be successfully reverted, we used machine learning (random forest ^43^) approaches to first identify metabolite features that differ between uninfected and infected untreated groups at our 142 day timepoint. We then assessed which of these features were restored by treatment (see Methods). Overall, we identified 27 to 64 metabolite features perturbed by infection at these cutoffs depending on the heart position. Metabolites altered by treatment were mainly site-specific, indicating local impacts of treatment (Fig. 4B). Concurring with our observations of minimal metabolic perturbation at the left ventricle top and right atrium, almost all infection-perturbed metabolites at these sites could be restored by at least one treatment (Fig. 4BC). In contrast, at the other heart positions, only about half of the infection-perturbed metabolites were restored by any treatment (compare Fig. 4B to Fig. 4A; **Supplementary table 3, Supplementary data 3**). Several metabolites were commonly restored by both treatment regimens, likely due to the overlapping treatment composition (Fig. 4F). However, as expected based on its greater positive impact on overall metabolism (Fig. 3), the combination treatment restored more metabolites than BNZ-only treatment (Fig. 4D vs Fig. 4E). For example, *m/z* 720.445 RT 3.09 min (annotated as PC 28:3;O3) in the left ventricle top and *m/z* 396.332 RT 2.991 min (no annotation) in left ventricle bottom recovered closer to naive in mice that received the combination treatment than in mice that received BNZ only (Fig. 4G). In contrast, *m/z* 137.046 RT 0.35 min (annotated as hypoxanthine) in the right ventricle top and *m/z* 494.325 RT 2.865 min (annotated as LPC 16:1 or LPC O-16:2;O) in the left ventricle bottom recovered better in mice that received the BNZ treatment than in mice that received the combination treatment, though still incompletely. In aggregate, some metabolite classes were more readily restored than other metabolite classes. For example, the majority of infection-perturbed lipid and lipid-like molecules were restored by treatment, except in the left ventricle bottom. In contrast, nucleosides and nucleoside analogs were predominantly not-restored (Table 2, Fig. 4G).

Given the availability of time-course tissue metabolomic data, we then tested the hypothesis that metabolites already perturbed at early infection timespoints (50 days post-infection or 75 days post-infection) might be harder to restore by treatment. However, we did not observe a clear temporal association pattern between metabolites that were restored by treatment and those that were not (Table 3).

### Stronger association between metabolic features that are not restored by treatment and disease parameters

**Figure 5.**
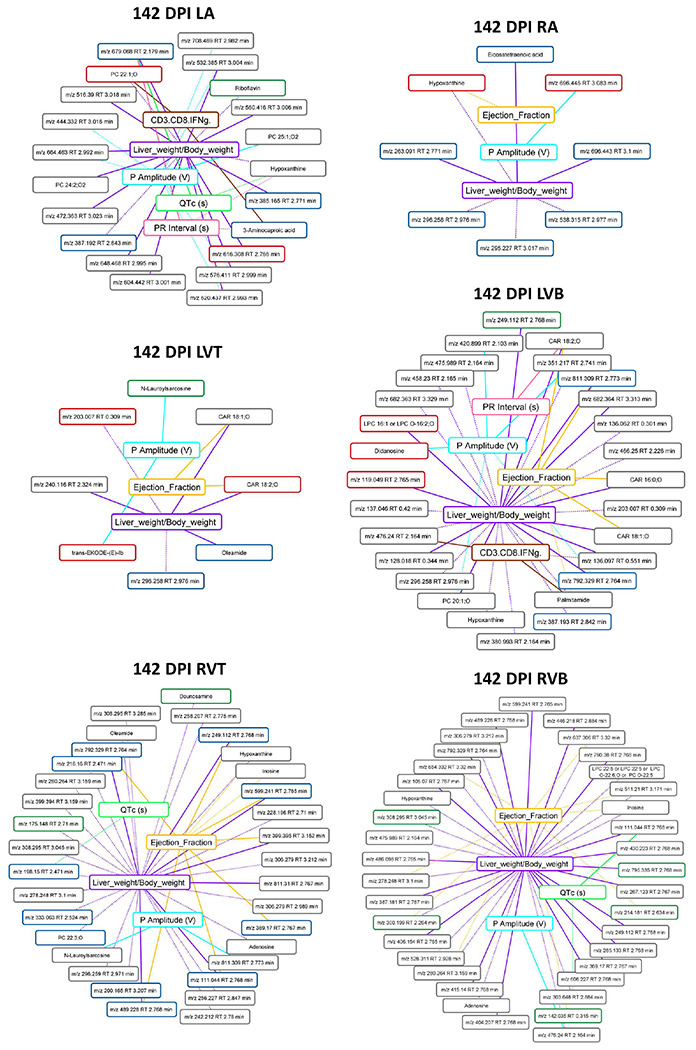
Correlation between disease metadata and metabolite peak area at 142 DPI. Solid lines indicate a positive correlation coefficient between disease metadata and metabolites; dotted lines indicate a negative correlation coefficient between disease metadata and metabolites. Correlation line colors relate to the specific correlated metadata category. Metabolite box colors indicate feature restoration status: green for features restored by combo treatment, red for features restored by BNZ treatment, blue for features restored by both treatments, and grey for features not restored by any treatment. Note the greater number of not-restored features correlated to the metadata.

We then sought to determine whether specific metabolites that failed to be restored by treatment could be associated with persistent disease symptoms. Given the overlap between some of these parameters (e.g. ejection fraction and stroke volume) and the high likelihood of common biological interdependence (e.g. between IL2 and IFNγ), we therefore first assessed whether each of the metadata parameters were correlated with each other. This analysis identified seven sets of strongly co-correlated or anti-correlated parameters (**Supplementary Fig. 1**), from which we chose one representative parameter from each category for downstream analysis. We then assessed whether restored vs not-restored metabolite features correlated with these parameters (**Supplementary data 2**). Following multiple hypothesis correction, a significantly greater proportion of the not-restored metabolites were significantly correlated with these metadata parameters in the ventricle segments (Table 4, Fisher’s exact test, p<0.05; Fig. 5). Likewise, the maximal absolute correlation strength was overall greater for more metadata categories for the not-restored metabolite features (**Supplementary data 2**), indicating that our observation is not an artefact of the multiple hypothesis correction. This was particularly apparent for correlations to liver weight normalized to body weight in the left atrium, left ventricle bottom, right ventricle top, and right ventricle bottom. Additional correlations were also observed with P wave amplitude, another disease parameter that failed to be restored by either treatment, at all sampling sites. Correlations with ejection fraction were also commonly observed, at all sites except the left atrium.

**Figure 6.**
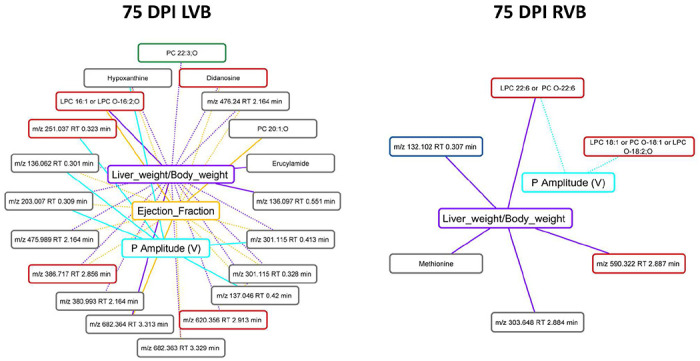
Correlation between disease metadata and metabolite peak area at 75 DPI. Solid lines indicate a positive correlation coefficient between disease metadata and metabolites; dotted lines indicate a negative correlation coefficient between disease metadata and metabolites. Correlation line colors relate to the specific correlated metadata category. Metabolite box colors indicate feature restoration status: green for features restored by combo treatment, red for features restored by BNZ treatment, blue for features restored by both treatments, and grey for features not restored by any treatment. Note the greater number of not-restored features correlated to the metadata in LVB.

This relationship was already apparent at 75 days post-infection (pre-treatment) in the left ventricle bottom, where 16% of features restored by treatment were significantly correlated with metadata parameters, compared to 50% of not-restored features (Fisher’s exact test, p=0.0109, Fig. 6). In contrast, in the right ventricle bottom at 75 days post-infection, there were comparable numbers of restored and not-restored features correlated with metadata (Fisher’s exact test, p>0.05, Fig. 6). These observations suggest inherent biological differences between restored and not-restored metabolite features, including a tighter relationship between not-restored features and disease indicators. In contrast, there was no clear relationship between the proportion of features restored by Bz-only treatment or the combination treatment correlated to metadata across sampling sites and timepoints (**Supplementary data 2**).

## Discussion

The ultimate goal of any therapeutic regimen is to return the patient to their pre-disease health state or better. In the context of infectious diseases, drug development has primarily focused on clearance of the pathogenic agent. However, there are increasingly frequent reports of conditions where this is insufficient to alleviate all patient symptoms (*e.g.*^1,2,3,5^). Thus, next-generation drug development necessitates a better understanding of the recovery processes following infection and treatment. We addressed these questions in the context of *T. cruzi* infection over time and following treatment with either the standard-of-care antiparasitic BNZ ^56^ , or an experimental combination of BNZ and Tc24-C4/E6020-SE therapeutic vaccine, with a focus on infection-induced metabolic alterations. We elected to focus on metabolism given its extensive association with cardiac function ^57^_^59^, our findings of correlations between disease severity and degree of metabolic perturbation in chronic CD ^32^, and our prior report of improved infection outcome via metabolic modulation in acute CD, independent of parasite burden ^33^. Given the low parasite load during chronic *T. cruzi* infection, detected metabolites are most likely host-derived. Metabolic changes therefore reflect both direct parasite impacts on host metabolism, as well as host compensatory mechanisms and immunometabolism ^60,61^.

Timecourse analysis revealed divergent metabolic trajectories depending on cardiac regions (Fig. 1BC), with the greatest metabolic perturbations in chronic-stage disease at apical cardiac segments (Fig. 1BC), confirming in an independent infection model our prior findings^31^ and concurring with clinical observations of apical aneurysms in CD patients ^53,56^ . Furthermore, the observed disconnect between sites of metabolic alterations and sites of high parasite load (Fig. 1B vs Fig. 3B) concurs with our prior observation in cardiac and gastrointestinal tissues ^31,33^ and *in vitro* ^62^, and the low parasite burden sometimes disconnected from lesion sites in human CD patients ^56,63–65^.

Strikingly, we observed that, although BNZ treatment successfully cleared the cardiac parasite burden (Fig. 3BF), this only restored half of the phenotypic indicators of disease (Table 1) and was insufficient to fully restore cardiac metabolism in the left ventricle bottom, right ventricle bottom and right ventricle top (Fig. 3CD, **Supplementary data 1**). Jointly, these findings provide a mechanistic metabolic model to explain the failure of BNZ treatment to improve cardiac outcomes in late-stage CD patients in the BENEFIT clinical trial ^5^. In contrast, BNZ shows better efficacy clinically when treatment is initiated earlier following infection ^56^. We likewise observed good metabolic restoration with acute-stage BNZ treatment ^33^, or in this study in the chronic stage at sites such as the right atrium and left ventricle top that were less metabolically perturbed. This may reflect greater tissue metabolic resilience if the parasite is rapidly cleared or cleared prior to the establishment of broad metabolic alterations. However, at the individual metabolite level, we did not observe a clear association between duration of individual metabolite perturbation and ease of individual metabolite restoration (Table 3).

Purines were particularly poorly restored by treatment as a class overall. Given that BNZ treatment clears parasite load, this cannot be due to current parasite purine scavenging ^66^, though could reflect prior and incompletely restored parasite-mediated depletion. Treatment of CD patients with allopurinol, a purine catabolism inhibitor, failed to reduce parasite burden ^67^ but was associated with re-normalization of cardiac function ^68^, and may thus be worth re-examining in combination with existing or experimental antiparasitic agents based on our findings.

Encouragingly, combining BNZ with the Tc24-C4/E6020-SE therapeutic vaccine provided greater immunological and metabolic benefits than BNZ-only treatment, even though parasite clearance was less than with the high-dose BNZ-only treatment (Table 1, Fig. 3B–F). This indicates opportunities for clinical dose-reduction of BNZ when implemented as part of a combination treatment regimen, which may help lessen treatment adverse effects ^55,69^. In combination with our timecourse findings and prior work by us and colleagues ^31,33,70–72^ , these results demonstrate that successful CD treatment will necessitate not only parasite clearance, but also the lessening of host tissue dysfunction, and may indicate a need to redefine the CD target product profile for therapeutics and for assessment of treatment efficacy ^27–29,73,74^ . Our metabolomic approach also provides a method to test new interventions for their superiority to BNZ-only treatment prior to clinical implementation, as demonstrated here with BNZ+Tc24-C4/E6020-SE vaccine combination treatment.

One limitation of this study is the fact that we could not confirm sterile cure via immunosuppression, due to the need to collect cardiac tissue for metabolomics. Thus, it is possible that the persistent metabolic alterations in the BNZ treatment group may be due to low-level parasite persistence below our detection limit or regular cardiac re-invasion from gastrointestinal reservoirs ^37^ , which were not analyzed here. However, this is unlikely to be the dominant cause of metabolic perturbations, as improved metabolism is observed in the BNZ+Tc24-C4/TLR4 agonist group even though parasite burden is higher. These results also demonstrate that persistent metabolic alterations post-BNZ treatment are not from leftover parasite fragments (unlike what is observed in post-infectious Lyme arthritis^75^, for example). Instead, given that only the combination treatment and not BNZ-only treatment restored CD3^+^CD8^+^TNFa^+^, CD3^+^CD8^+^IFNg^+^ and CD3^+^CD8^+^IL.2^+^ cell levels in our study, this may reflect persistent immunological imbalance that can only be restored by an immunotherapeutic and not by BNZ-alone. Prior work on BNZ treatment showed improvement of infection-induced immunological perturbations ^76^, but with incomplete IFNγ restoration. Indeed, three of the metabolite features that failed to be restored by treatment in the left ventricle bottom were correlated with CD3^+^CD8^+^IFNg^+^ cell levels. Active follow-up work in our laboratory is assessing the direct relationship between IFNγ levels and CD cardiac metabolic alterations.

Our approach also does not provide cellular-level insight into hyperlocal processes, even though these are clearly also important to CD pathogenesis ^77,78^. Single-cell metabolomics of *in vitro T. cruzi* infection have demonstrated bystander effects of infection on infection-adjacent but uninfected cells, as well as similar alterations to phospholipid metabolism compared to our chemical cartography analyses ^62^. These bystander effects on non-parasite-containing cells may help explain why parasite clearance is insufficient to improve metabolism.

A last, necessary limitation of our tissue analyses is that they cannot be performed sequentially on the same animals due to the invasive nature of the sample collection. Thus, each timepoint and treatment sample is derived from a different animal. While biofluids could have been sampled non-invasively, they do not necessarily reflect the metabolic changes occurring at the site of disease processes. Validation in humans would be desirable, though generating adequately controlled studies with short post-mortem interval to avoid effects on metabolism would be challenging. Our results do however concur with findings in human serum following treatment with the antiparasitic nifurtimox, which likewise showed a lack of metabolic restoration in some patients in positive mode up to three years post-treatment ^79^. Longer-term follow-up studies should also be performed, though these are limited by the lifespan of the mouse model.

Overall, these results have major implications for CD drug development, providing a mechanism to explain prior clinical treatment failures as well as a path to assess the superiority of novel treatment regimens in pre-clinical animal models. Importantly, these results and our prior work during acute-stage treatment and *in vitro* infection ^33,62^ confirm a spatial disconnect between clinical outcomes and parasite burden that should inform CD drug development. Our method also shows broad potential to study other chronic infectious disease and post-treatment chronic sequelae.

## Figures and Tables

**Table 1.** Impact of treatments on infection-perturbed phenotypes.

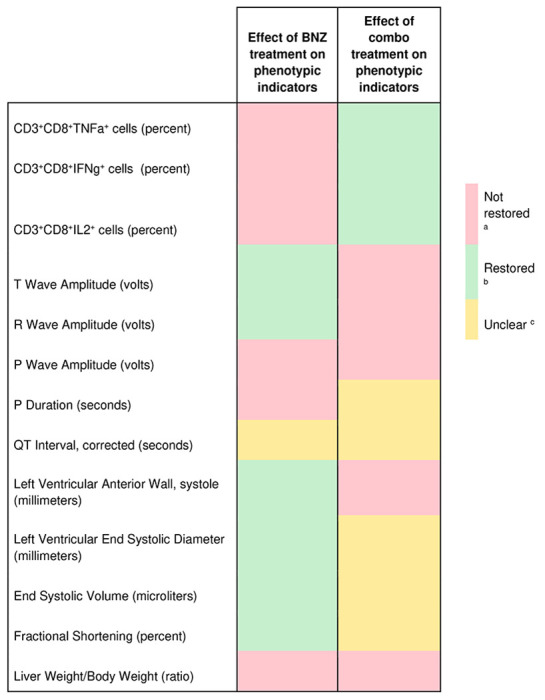

a FDR-corrected p<0.1 to naive group and FDR-corrected p>0.1 to infected untreated group

b FDR-corrected p<0.1 to infected untreated group and FDR-corrected p>0.1 to naive animals

c Not meeting criteria ^a^ or ^b^.

**Table 2.** Proportion of each metabolite superclass restored by treatment, per heart segment.

	Post-treatment status	LA	RA	LVT	LVB	RVT	RVB
Benzenoids	Restored	/ ^a^	/	**0**	/	/	/
	Not restored	/	/	1	/	/	/
Organic acids and derivatives	Restored	1	/	1	**0.5**	**0**	**0.5**
	Not restored	**0**	/	**0**	**0.5**	1	**0.5**
Lipids and lipid-like molecules	Restored	1	1	0.8	**0.25**	1	0.83
	Not restored	**0**	**0**	**0.2**	0.75	**0**	**0.17**
Organoheterocyclic compounds	Restored	**0**	1	/	**0**	0.67	**0**
	Not restored	1	**0**	/	1	0.33	1
Nucleosides, nucleotides, and analogues	Restored	/	1	/	**0.33**	**0**	**0.33**
	Not restored	/	**0**	/	0.67	1	0.67
	Restored	/	/	1	**0**	0.33	1
Phenylpropanoids and polyketides	Not restored	/	/	**0**	1	0.67	**0**
Organic nitrogen compounds	Restored	/	/	/	1	/	/
	Not restored	/	/	/	**0**	/	/
Organic oxygen compounds	Restored	/	/	/	/	/	/
	Not restored	/	/	/	/	/	/

a “/” means not perturbed by infection at this site.

b Values ≤0.5 in bold.

**Table 3.** Proportion of metabolites for each treatment response behavior that were significantly perturbed at early infection timepoints.

Timepoint	Sections	Restored by any treatment	Restored by BNZ+Tc24 combination	Restored by BNZ-only treatment	Restored by both treatments	Not restored
50 DPI	LA	0.42	1	0.33	0.38	0.84
	RA	0.1	No metabolites restored by BNZ+Tc24 combination	0	0.11	No metabolites that failed to be restored
	LVT	0.6	0.71	0.5	0.43	0
	LVB	0.76	0.73	0.83	0.75	0.72
	RVT	0.48	0.5	0	0.5	0.31
	RVB	0.07	0	0.25	0	0.03
75 DPI	LA	0	0	0	0	0
	RA	0.1	No metabolites restored by BNZ+Tc24 combination	0.33	0.07	No metabolites that failed to be restored
	LVT	0.12	0	0.5	0.14	0
	LVB	0.28	0.07	0.83	0.25	0.62
	RVT	0.16	0.12	0	0.18	0.28
	RVB	0.14	0	0.38	0.14	0.09

**Table 4.** Proportion of restored and not-restored metabolite features that were correlated to disease severity metadata (142 days post-infection).

Sections	Restored	Not restored	Fisher’s exact test
LA	58%	74%	Non-significant
RA	23%	All features were restored by treatment	Not applicable
LVT	16%	100%	p=0.0427
LVB	4%	73%	p<0.00001
RVT	35%	63%	p=0.0446
RVB	10%	91%	p<0.00001
